# Notes on Preparations for the Sick

**Published:** 1909-12

**Authors:** 


### Notes on Preparations for the Sick.

**Lactilloids.**—Ferris & Co., Bristol.—These tablets, from active cultures of the Bulgarian and other lactic acid bacilli, are prepared for use in accordance with the researches and views of Professor Metchnikoff. Curdled milk, and the various preparations of the bacilli which make it, are quite the most popular medication at the present time. It is beset with great expectations, and it is found that people who take it are none the worse, but often describe themselves as much better. The directions for use are:—

*For direct administration.*—One or two Lactilloids to be taken three times a day before meals in milk or water, sweetened with a little sugar, sugar of milk, or malt extract.

*For the preparation of Curdled Milk.*—Boil one pint of fresh milk for about fifteen minutes; allow it to cool to about 100° F., and add two or three Lactilloids. Keep at the same temperature for from eight to ten hours; the milk will then be ready for use. About five ounces of the curdled milk should be taken three times a day before food.

**"Tyramine."**—Burroughs, Wellcome & Co., London.—"Tyramine" presents in a state of purity the organic base p-hydroxyphenylethylamine, which has recently been shown by researches at the "Wellcome" Physiological Research Laboratories to be the chief active principle of aqueous extract of ergot. Given hypodermically or by the mouth, it produces a marked rise of blood-pressure, with greatly improved vigour of the heart's action. "Tyramine" may be administered in shock or collapse, and for producing contraction of the uterus post partum.

"Tabloid" Hypodermic "Tyramine," 0.005 gramme, is issued in tubes of twelve, ready for immediate use in emergency.

---

**Formenthoids.**—BUXTON & Co., Clifton.—These tablets are a pleasant combination of formaldehyde and menthol, with a suitable flavouring base. They should be sucked slowly for purposes of mouth and throat disinfection.

---

**Thymoform.**—OPPENHEIMER, SON & Co., London.—A combination in tablet form of thymol and formic aldehyde, with a cane sugar basis. They are very similar to the above-mentioned, and to the much advertised "Formamint" tablets.

---

**Aldoform.**—DUNCAN, FLOCKHART & Co., London.—Each of these tablets contains 1 per cent. of formaldehyde in combination with sugar of milk. Tablets such as these have been proved to be as effective in practice as they are in theory in tending towards oral asepsis.

---

**Methylanodyne: Unguentum Analgesicum.**—BURNETT AND TURNER, Clifton.—A readily absorbed preparation of methyl salicylate, menthol, oil of camphor, etc., with a lanoline base, dispensed in collapsible metal tubes to prevent the volatilisation of the more important ingredients.

The admixture of menthol with a considerable quantity of salicylate of methyl produces a most powerful analgesic for external use: its penetrative qualities and ready absorption by the skin recommend it, in conjunction with the internal use of the salicylates or other analgesics, in the treatment of acute or chronic rheumatism, gout, sciatica, pleurisy, and all neuralgic pains.

---

**Ferro-Glidine, Luesan, Arsan.**—MENLEY & JAMES, London.—We have previously commented on Glidine (p. 183), and its iodine and bromine combinations (p. 281). We have now to mention that similar compounds, with iron, mercury, and arsenic respectively, are now prepared in tablet form, and should be very efficient and useful preparations. The combination with an organic basis renders these drugs non-toxic, and the protein basis is itself of some value in the treatment of cases of tuberculosis and other diseases of malnutrition. The "Luesan" tablet contains 0.01 gramme of mercury in organic combination, and the "Arsan" contains 0.002 gramme of arsenic, likewise in combination with a vegetable protein food.

---

**Fer Ascoli.**—ALLEN & HANBURYS, London.—These tablets are essentially an organic compound of iron. They are described as a compound of iron with nuclein. They have proved themselves of value, more particularly in cases of chlorosis and anæmic dyspepsia, when the mineral preparations of iron are said to disagree. They should be taken with meals, and are believed to remain insoluble until they come into contact with the alkaline secretions of the intestine. We are told that they contain 8 per cent. of iron, and it is believed that the ingestion of the nuclein with which the iron is combined has the effect of producing a marked phagocytosis, and so increases the tissue resistance to bacterial invasion.

---

**Kephaldol.**—Fr. Stohr & Co., London.—" Kephaldol " may be regarded as a mild, promptly acting antipyretic, antineuralgic and antihidrotic, that does not produce any undesirable and injurious by-effects on the various organs.

It is a yellowish-white powder (also put up in 7½ gr. tablets), with a somewhat bitter taste. It is but slightly soluble in water, ether and chloroform, comparatively readily so in alcohol. " Kephaldol " is a product of reaction, resulting through the action by citric acid and salicylic acid on various forms of phenetidine, at the end of which any free acid still present is fixed to quinine, or neutralised by sodium carbonate, respectively.

Various German authors have given glowing accounts of the satisfactory results obtained by this drug.

---

**Antineurasthin.**—The Antineurasthin Co., London.—The name of this drug does not commend it to us. The drug is accompanied by a little pamphlet, describing our nervous generation, the symptoms of nervous debility, and the modes and forms of neurasthenia. It then goes on to describe Dr. Hartmann's Brain and Nerve Food. We are not told what it is and how it is made, but it is described as a nutritive preparation characterised by its particularly high contents of Lecithine, and consequently highly adapted for use as a nerve food. Chemical examination yielded the following results :—

| | |
|---|---|
| Water .. .. .. .. .. .. | 4·75 |
| Mineral Substances .. .. .. .. | 2·33 |
| Ether Extract .. .. .. .. .. | 13·75 |
| Nitrogen .. .. .. .. .. .. | 3·17 |
| Nitrogenous Substances .. .. .. | 19·75 |
| Carbo-hydrates and Non-nitrogenous Extractives | 58.42 |
| Lecithine .. .. .. .. .. .. | 8.80 |

It is presumed to be adapted for all persons who are liable to mental stress and strain and to over-stimulation of the nerves, in particular for neurasthenics of every kind, and convalescents. As a rule, one tablet three times a day should suffice.

